# Protein Kinase N2 Reduces Hydrogen Peroxide-inducedDamage and Apoptosis in PC12 Cells by AntiOxidative Stress and Activation of the mTOR Pathway

**DOI:** 10.1155/2022/2483669

**Published:** 2022-09-21

**Authors:** Lin Wang, Lin Zhang

**Affiliations:** ^1^Department of Orthopedics, Yijishan Hospital, Wannan Medical College, Wuhu 241000, Anhui, China; ^2^Hangzhou TCM Hospital Affiliated to Zhejiang Chinese Medical University, Hangzhou 310053, Zhejiang, China

## Abstract

**Objective:**

To investigate the role and mechanism of protein kinase N2 (PKN2) in hydrogen peroxide (H_2_O_2_)-induced injury of PC12 cells.

**Method:**

s. PC12 cells were transfected with lentivirus to knock down or overexpress PKN2 and then were treated with 300 *μ*M H_2_O_2_ to establish a cell model of oxidative stress injury. The cell viability of PC12 cells in each group was determined by the CCK-8 method. Biochemical assays were used to measure reactive oxygen species (ROS), malondialdehyde (MDA) levels, and superoxide dismutase (SOD) activity. Western blot was used to detect the protein expressions of PKN2, caspase-3, cleaved-caspase-3, PARP, cleaved-PARP, p-mTOR, and mTOR in PC12 cells in each group.

**Results:**

H_2_O_2_ treatment could significantly reduce PC12 cell viability and promote cell apoptosis and oxidative stress. PKN2 overexpression inhibited H_2_O_2_-induced apoptosis and oxidation damage by increasing PC12 cell viability, SOD activity, and p-mTOR protein expression, reducing intracellular ROS and MDA levels, and cleaved-caspase-3 and cleaved-PARP protein expression.

**Conclusion:**

PKN2 overexpression can alleviate H_2_O_2_-induced oxidative stress injury and apoptosis in PC12 cells by activating the mTOR pathway.

## 1. Introduction

Many central nervous system diseases, such as cerebral ischemia, spinal cord injury, Alzheimer's disease, Parkinson's disease, amyotrophic lateral sclerosis, Huntington's disease, and so on, often show neuron injury and death [1, 2]. These neurological disorders share common risk factors such as aging, oxidative stress, environmental stress, and protein dysfunction [3]. Oxidative stress damages the integrity of neurons causes cell necrosis or apoptosis and causes damage to the structure and function of the nervous system [4]. Since oxidative stress is a promising therapeutic target for nervous system disease treatments.

Protein kinase N (PKN) is a subfamily of AGC serine/threonine protein kinase. It consists of three subtypes, PKN1, PKN2, and PKN3. Because of its extensive biological functions, such as regulating the cell cycle, receptor transport, vesicle transport, cell apoptosis, and so on, it has attracted more and more attention [5]. PKN2, a member of the PKN2 family, has been found to promote axon growth and play an important role in the migration of neural crest in mouse mesodermal development [6, 7]. However, whether PKN2 has a protective effect on nerve cells and what mechanism is involved in this protective effect is not clear. Mammalian rapamycin (mTOR) is widely distributed in the central nervous system, which can promote the proliferation, differentiation, and survival of nerve cells and regulate synaptic plasticity [8]. PC12 cells are a recognized neuronal cell model for neuronal mechanistic studies and the detection of potentially neurotoxic substances [9]. In this study, the oxidative stress model of rat pheochromocytoma (PC12) cells induced by H_2_O_2_ was used to investigate whether PKN2 has a protective effect on the H_2_O_2_-induced PC12 cell injury model and the possible mechanism of PKN2 and mTOR pathway.

## 2. Materials and Methods

### 2.1. Cell Culture

PC12 cells were purchased from the Shanghai Institute of life sciences, Chinese Academy of Sciences. PC12 cells were cultured in Dulbecco's Modified Eagle Medium (DMEM, Gibco, USA) containing 10% fetal bovine serum (FBS, Gibco, USA) and 1% penicillin-streptomycin (Gibco, USA) at 37°C, 5% of CO_2_ incubator.

### 2.2. Cell Transfection and Grouping

Negative control lentivirus and PKN2 shRNA lentivirus, PKN2 overexpression lentivirus, and vector lentivirus were designed and synthesized by Wuhan University (sequence number: br005591). The RNAi target sequence was ACGCTCGGGTGATGTTKATTA, and the negative control target sequence was ttctccgaacgtcacgt. PC12 cells in the logarithmic growth phase were selected and transfected using Lipofectamine™ 3000 Transfection Reagent (Invitrogen) according to the manufacturer's instructions, and cells were grouped into control group, siNC group, PKN2i group, vector group, and PKN2-OE group. 72 h after transfection, the expression levels of PKN2 in each group of cells were detected by western blot.

### 2.3. Construction of Oxidative Stress Model

The transfected PC12 cells were treated with or without H_2_O_2_ (300 *μ*M) for 8 h to induce an oxidative stress model [10, 11]. The transfected PC12 cells were divided into the control group, model group (H_2_O_2_), siNC + H_2_O_2_ group, PKN2i + H_2_O_2_ group, vector + H_2_O_2_ group, and PKN2-OE + H_2_O_2_ group. When the cells grow to a certain number, the cells are collected.

### 2.4. Cell Viability Assay

Cell viability was detected by the Cell Counting Kit-8 (CCK-8) assay. Transfected PC12 cells were seeded in a 96-well plate at 1 × 10^4^ cells/well, 100 *μ*L per well. Cell grouping and drug treatment were as described above. After 24 h, the medium was replaced with a DMEM medium containing 10 *μ*L of CCK-8 solution (Beyotime), and the incubation was continued for 2 h. Then, the absorbance at 450 nm was measured using a microplate reader (BioTek Instruments).

### 2.5. Intracellular Oxygen Species (ROS), Malondialdehyde (MDA), and Superoxide Dismutase (SOD) Measurement

PC12 cells were seeded in 6-well plates at 1 × 106 cells/mL, and the cell supernatants were collected after cell grouping and administration as described above. Intracellular ROS, MDA, and SOD levels were determined using a ROS assay kit, lipid peroxidation assay kit, and SOD assay kit following the manufacturer's instructions (Nanjing Jiancheng).

### 2.6. Western Blot

PC12 cells were seeded in a 6-well plate at 1 × 10^6^ cells/mL, and the cells were grouped and treated as described above. Cells were harvested, and total cell protein was extracted using RIPA lysis buffer (Solarbio) containing PMSF and phosphatase inhibitors, and the protein concentration was determined using a BCA protein detection kit (Beyotime). Protein samples were separated by SDS-PAGE electrophoresis, and proteins were transferred into a polyvinylidene fluoride (PVDF) membrane. Membranes were blocked with 5% skim milk or BSA for 1 h at room temperature. Then, the membranes were mixed with anti-PKN2-antibody, anticaspase-3-antibody, anticleaved-caspase-3-antibody, anti-PARP-antibody, anticleaved-PARP-antibody, anti-mTOR-antibody, anti-p-mTOR-antibody, and anti-GAPDH-antibody (all primary antibodies were purchased from Abcam, using ratio 1 : 1000) were incubated overnight. The next day, wash the membrane with TBST, then incubate the membrane with anti-IgG secondary goat anti-mouse antibody (1 : 5000, Abcam) or anti-IgG goat anti-rabbit antibody (1 : 5000, Abcam) at room temperature for 1h. The protein bands were displayed using the BeyoECL Star kit (Beyotime, China), and the gray values of the protein bands were determined by Image-Pro Plus software.

### 2.7. Statistical Analysis

SPSS 20.0 and GraphPad Prism 9.0 software were used for statistical analysis and visualization of experimental data. Comparisons between multiple groups were performed using one-way ANOVA, and differences between two groups were analyzed using Student's *t*-test. The experimental results are expressed as mean ± standard deviation (SD). *P* < 0.05 was considered a statistically significant difference.

## 3. Results

### 3.1. PKN2 Overexpression is Protective against H_2_O_2_-induced PC12 Cells

**Figure 1:**
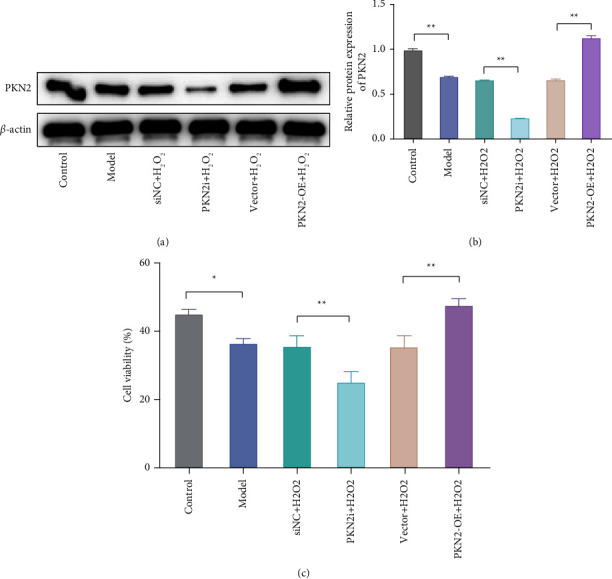
PKN2 overexpression has a protective effect on H_2_O_2_-induced PC12 cells. (a/b) PKN2 protein expression in PC12 cells after transfection were detected by western blot. (c) Effects of PKN2 knockdown or overexpression on H_2_O_2_-induced PC12 cell viability. ^*∗∗*^*P* < 0.01*vs*. control, siNC + H_2_O_2_, and vector + H_2_O_2_.

To investigate the effect of PKN2 on oxidative damage in PC12 cells. First, we knocked down or overexpressed PKN2 in PC12 cells by transfection and detected the transfection efficiency by western blot. Compared with the Si-NC group, PKN2 protein expression in the PKN2i group was significantly decreased, and compared with the vector group, the PKN2 protein expression in the PKN2-OE group was significantly increased (Figures 1(a), 1(b)).

Subsequently, the effect of PKN2 on H_2_O_2_-induced PC12 cell viability was detected by the CCK8 assay. The results showed that compared with the control group, the viability of PC12 cells was significantly reduced after H_2_O_2_ treatment, indicating that the oxidative damage model of PC12 cells was successfully established. Further analysis showed that compared with the siNC + H_2_O_2_ group, the cell viability of the PKN2i + H2O_2_ group was significantly reduced. Compared with the vector + H_2_O_2_ group, the cell viability in the PKN2-OE + H_2_O_2_ group was significantly increased (Figure 1(c)). These results suggest that PKN2 overexpression can alleviate the toxic effects of H_2_O_2_ on PC12 cells.

### 3.2. PKN2 Overexpression Reduces H2O2-induced Oxidative Damage in PC-12 Cells

**Figure 2:**
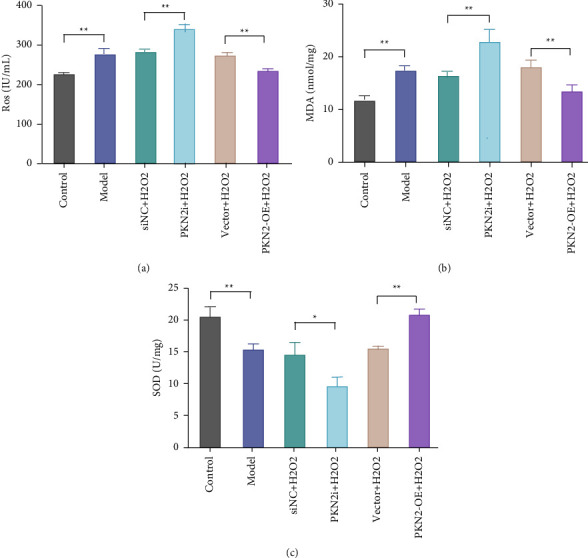
PKN2 overexpression reduces H_2_O_2_-induced oxidative damage in PC12 cells. (a–c) Quantitative analysis of intracellular levels of ROS, (a) MDA, and (b) relative activity of SOD in each group. ^*∗∗*^*P* < 0.01*vs*. control, siNC + H2O_2_, and vector + H_2_O_2_.

The production of ROS and MDA and the changes in SOD activity are important markers of oxidative stress in cells [12]. Previous studies have shown that ROS is an important mediator of H_2_O_2_-induced cell death [13]. To explore the role of PKN2 in H_2_O_2_-induced oxidative stress in PC12 cells, we examined the production of ROS and MDA and the activity of SOD. The results showed that in PC12 cells, compared with the control group, the levels of ROS and MDA in the cells of the model group were significantly increased, and the activity of SOD was significantly decreased. Compared with the siNC + H_2_O_2_ group, knockdown of PKN2 could significantly increase the levels of ROS and MDA and decrease the activity of SOD. Compared with the vector + H_2_O_2_ group, PKN2 overexpression significantly decreased the levels of ROS and MDA in PC12 cells and increased the activity of SOD (Figures 2(a)–2(c)). The above results indicate that overexpression of PKN2 could significantly inhibit the oxidative damage of H_2_O_2_ on PC12 cells and exert an antioxidative stress effect.

### 3.3. PKN2 Overexpression Prevents H_2_O_2_-induced Apoptosis in PC12 Cells

**Figure 3:**
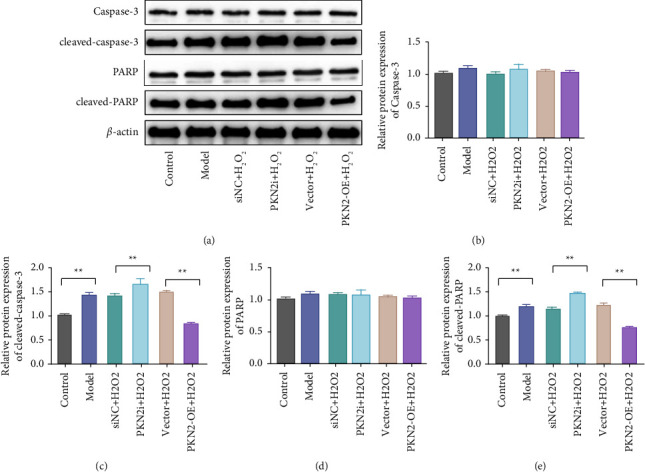
PKN2 overexpression prevents H_2_O_2_-induced apoptosis of PC12 cells. (a) The protein expression levels of PARP, cleaved PARP, caspase-3, and cleaved caspase-3 in cells of each group were detected by western blot. (b–e) Image-Pro Plus software to analyze the gray values of PARP, cleaved PARP, caspase-3, and cleaved-caspase-3 proteins in each group of cells ^*∗∗*^*P* < 0.01*vs*. control, siNC + H_2_O_2_, and vector + H_2_O_2_.

The excessive accumulation of ROS caused by the dysfunction of mitochondria in cells is an important inducement for apoptosis [14]. Therefore, we further explore the effect of PKN2 on H_2_O_2_-induced apoptosis in PC12 cells. The results showed that compared with the control group, the expression of cleaved-PARP and cleaved-caspase-3 proteins in the cells of the model group was significantly increased. Compared with the siNC + H_2_O_2_ group, knockdown of PKN2 significantly increased the expression levels of cleaved-PARP and cleaved-caspase-3. Compared with the vector + H_2_O_2_ group, PKN2 overexpression could significantly reduce the expressions of cleaved-PARP and cleaved-caspase-3 proteins. In addition, PARP and caspase-3 expressions did not change significantly in each group of cells (Figures 3(a)(a)–3(e)). These results suggest that PKN2 overexpression may inhibit H_2_O_2_-induced apoptosis in PC12 cells by reducing the production of oxidative stress.

### 3.4. PKN2 Overexpression Inhibits H_2_O_2_-induced Apoptosis in PC12 Cells by Activating the mTOR Pathway

**Figure 4:**
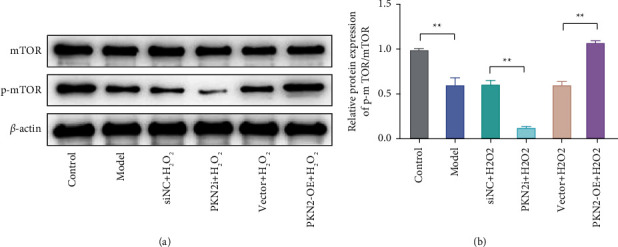
PKN2 overexpression inhibits H_2_O_2_-induced apoptosis in PC12 cells by activating the mTOR pathway. (a–b) Western blot detection of mTOR and p-mTOR protein expression and p-mTOR/mTOR ratio in cells of each group. ^*∗∗*^*P* < 0.01*vs*. control, siNC + H_2_O_2_, and vector + H_2_O_2_.

Studies have shown that the Mammalian target of rapamycin (mTOR) plays an important role in regulating autophagy and apoptosis, and activation of mTOR can alleviate ROS-mediated ER stress-induced apoptosis of CD_4_ T cells [15]. In the present study, the mechanism of PKN2 on H_2_O_2_-induced apoptosis in PC12 cells were investigated by detecting mTOR pathway-related proteins. The results showed that the expression of p-mTOR protein and the ratio of p-mTOR/mTOR in the cells of the model group were significantly lower than those of the control group. Compared with the siNC + H_2_O_2_ group, knockdown of PKN2 could significantly reduce p-mTOR protein expression and p-mTOR/mTOR ratio. Compared with the vector + H_2_O_2_ group, PKN2 overexpression significantly increased p-mTOR protein expression and p-mTOR/mTOR ratio. At the same time, there was no significant change in the expression level of mTOR protein in the cells of each group (Figures 4(a)-4(b)). These results suggest that PKN2 overexpression may reduce H_2_O_2_-induced apoptosis in PC12 cells by activating the mTOR pathway.

## 4. Discussion

Diseases of the central nervous system often manifest as neuronal death. There is increasing evidence that oxidative stress is important pathogenesis of many central nervous system diseases [16–18]. Therefore, inhibiting oxidative stress can reduce neuronal damage, which is of positive significance for the prevention and treatment of neurological diseases. PC12 cells have been widely used in the study of neurological diseases [19]. In addition, H_2_O_2_, as a precursor of reactive oxygen species and reactive nitrogen species, can easily pass through biofilms and enter cells, thus, it has long been used as a stimulator to induce oxidative stress models [20, 21]. Therefore, in this study, PC12 cells were stimulated with H_2_O_2_ to induce an oxidative stress model to explore the effect of PKN2 on neuronal death and its mechanism.

ROS are a normal by-product of aerobic metabolism in eukaryotic cells. Low to moderate concentrations of ROS are involved in immune responses, signal transduction, and other processes under physiological conditions. However, excessive ROS production may cause oxidative damage to cellular biomolecules such as proteins, lipids, and nucleic acids [22]. Mammalian cells possess a variety of antioxidants and other cytoprotective factors that protect them from ROS damage [23]. SOD is the first-line antioxidant enzyme in organisms that catalyzes the conversion of superoxide to oxygen and hydrogen peroxide [24]. The generated hydrogen peroxide is converted into oxygen and water by catalase, thereby reducing the concentration of ROS [25]. When there is an imbalance between ROS production and degradation, excessive accumulation of ROS can lead to oxidative stress in cells, causing cell death. MDA is a relatively stable product of ROS attack on polyunsaturated fatty acids. Its content indirectly reflects the changes in intracellular oxygen-free radical content and the degree of lipid damage [26]. In this study, when PC12 cells were stimulated by H_2_O_2_, the levels of intracellular MDA and ROS were increased, and the cell viability and SOD activity were decreased. It can reduce H_2_O_2_-induced oxidative damage in PC12 cells and exert an antioxidative stress effect. Overexpression of PKN2 can significantly reduce the levels of MDA and ROS and increase the activity of intracellular SOD and cell viability, indicating that PKN2 overexpression can alleviate H_2_O_2_-induced oxidative damage in PC12 cells and play an antioxidative stress role. Mitochondria are the main site of reactive oxygen species production. H_2_O_2_ is a key reactive oxygen species produced by endogenous pathways in mitochondria [27]. When mitochondria are dysfunctional, excessive H_2_O_2_ can trigger the mitochondrial apoptosis pathway and eventually lead to apoptosis [28]. Due to its broad cytotoxicity to almost all cell types, H_2_O_2_ is currently the most widely used inducer to study apoptosis [29]. Consistent with previous findings [30–32], in the present study, caspase-3 was activated upon H_2_O_2_-induced apoptosis in PC12 cells, and the activated caspase-3 led to the cleavage of poly-ADP-ribose polymerase (PARP)-1, thereby triggering apoptosis. Therefore, cleaved caspase-3 and PARP-1 are often used as important markers for judging cell apoptosis [33, 34]. However, the overexpression of PKN2 not only inhibited the activation of caspase-3 but also inhibited the cleavage of PARP by caspase-3, and finally protected PC12 cells from H_2_O_2_-induced apoptosis.

mTOR is phosphorylated and activated by phosphatidylinositol 3-kinase (PI3K)/protein kinase B (Akt) in the canonical pathway and plays a role in inhibiting apoptosis and promoting cell survival [35]. Khallaghi et al. found that dimethylamine protects against oxidative stress in H_2_O_2_-induced PC12 cell injury by activating the mTOR signaling pathway [36]. In addition, animal experimental studies have shown that activation of the mTOR pathway is beneficial for reducing ischemia-reperfusion injury in rats by further inhibiting the process of inflammation, apoptosis, and oxidative stress [37]. In the present study, we found that PKN2 overexpression activates the mTOR pathway in PC12 cells to reduce H_2_O_2_-induced oxidative damage and apoptosis. It indicated that PKN2 may play an antioxidative damage and apoptosis effect by activating the mTOR pathway.

In conclusion, PKN2 participated in H_2_O_2_-induced oxidative stress injury by activating the mTOR signaling pathway, and its mechanism involves the regulation of mTOR protein phosphorylation. This study provides a reference for the study of the molecular mechanism of nerve injury and provides a potential new therapeutic target for the treatment of nervous system diseases. Because our experiment only explored mTOR signal pathway in PC12 cells, did not explore other signal pathways that PKN2 may play a role, and did not verify in primary nerve cells and animals, more research still needs to be further explored later.

## 5. Conclusion

In conclusion, PKN2 overexpression can alleviate the H_2_O_2_-induced PC12 cell damage via increasing cell viability and inhibiting cell apoptosis and oxidative stress. Further mechanism research showed that its protective effect in H_2_O_2_-induced PC12 cells may be related to the activated mTOR signaling pathway in PC12 cells.

## Data Availability

The data used to support the findings of this study are available from the corresponding author upon request.
